# Planar cell polarity in the larval epidermis of *Drosophila* and the role of microtubules

**DOI:** 10.1098/rsob.200290

**Published:** 2020-12-09

**Authors:** Stefano Pietra, KangBo Ng, Peter A. Lawrence, José Casal

**Affiliations:** Department of Zoology, University of Cambridge, Downing Street, Cambridge CB2 3EJ, UK

**Keywords:** planar cell polarity, microtubules, *dachsous*, *fat*, *ovo*, *dachs*

## Abstract

We investigate planar cell polarity (PCP) in the *Drosophila* larval epidermis. The intricate pattern of denticles depends on only one system of PCP, the Dachsous/Fat system. Dachsous molecules in one cell bind to Fat molecules in a neighbour cell to make intercellular bridges. The disposition and orientation of these Dachsous–Fat bridges allows each cell to compare two neighbours and point its denticles towards the neighbour with the most Dachsous. Measurements of the amount of Dachsous reveal a peak at the back of the anterior compartment of each segment. Localization of Dachs and orientation of ectopic denticles help reveal the polarity of every cell. We discuss whether these findings support our gradient model of Dachsous activity. Several groups have proposed that Dachsous and Fat fix the direction of PCP via oriented microtubules that transport PCP proteins to one side of the cell. We test this proposition in the larval cells and find that most microtubules grow perpendicularly to the axis of PCP. We find no meaningful bias in the polarity of microtubules aligned close to that axis. We also reexamine published data from the pupal abdomen and find no evidence supporting the hypothesis that microtubular orientation draws the arrow of PCP.

## 1. Introduction

As cells construct embryos and organs they need access to vectorial information that informs them, for example, which way to migrate, divide, extend axons and orient protrusions such as hairs. Cells in epithelia may produce oriented structures such as hairs or cilia and these are coordinated, pointing or beating in a particular orientation. This kind of polarity is known as planar cell polarity (PCP). In *Drosophila* there are (at least) two conserved genetic systems that generate PCP. Both systems rely on the formation of intercellular bridges made by transmembrane proteins containing cadherin repeats, these interact via their extracellular domains. The Dachsous/Fat (Ds/Ft) system depends on heterodimers of the protocadherins Ds and Ft while the Starry Night/Frizzled system relies on asymmetric homodimers of Starry Night (reviewed in [1–6]). Most developmental models can be tricky to study because both PCP systems operate at once and both have separate but confounding inputs into the orientation of bristles, etc. However, here we investigate the later-stage larvae in which PCP depends entirely on the Ds/Ft system [7–9], whose mechanism is quite well understood. Ds molecules in one cell bind to Ft molecules in a neighbour cell to make intercellular bridges. Experiments argue that, using the disposition and orientation of Ds-Ft bridges, each cell compares the Ds activity of those two of its neighbours that lie in the relevant axis and points its denticles towards the neighbour with the higher Ds activity. Ds activity is thus an important component of the model: the activity of Ds in a cell defines its ability to bind to Ft in its neighbouring cell, that activity depending on at least three factors; the levels of Ds expression, the levels of Ft expression and the activity of Four-jointed (Fj). Fj is a Golgi-resident kinase that phosphorylates both Ds and Ft, reducing the activity of the former while increasing the activity of the latter [10–12].

The system has an additional property: because of the interdependence of membrane bound Ds and Ft in neighbouring cells, the polarity of one cell can affect the polarity of its neighbours and that polarity can be propagated to the next neighbour [7,13,14]. Thus, in these several ways the landscape of Ds activity in a field of cells is translated into the individual polarities of the cells (see [5] for further explanation). More recently, we have, via experiments and observations, developed a model that explains the quite complex pattern of denticle polarities in the larval abdominal segment [15].

### 1.1. A model: the ventral epidermis of the *Drosophila* larva

**Figure 1.**
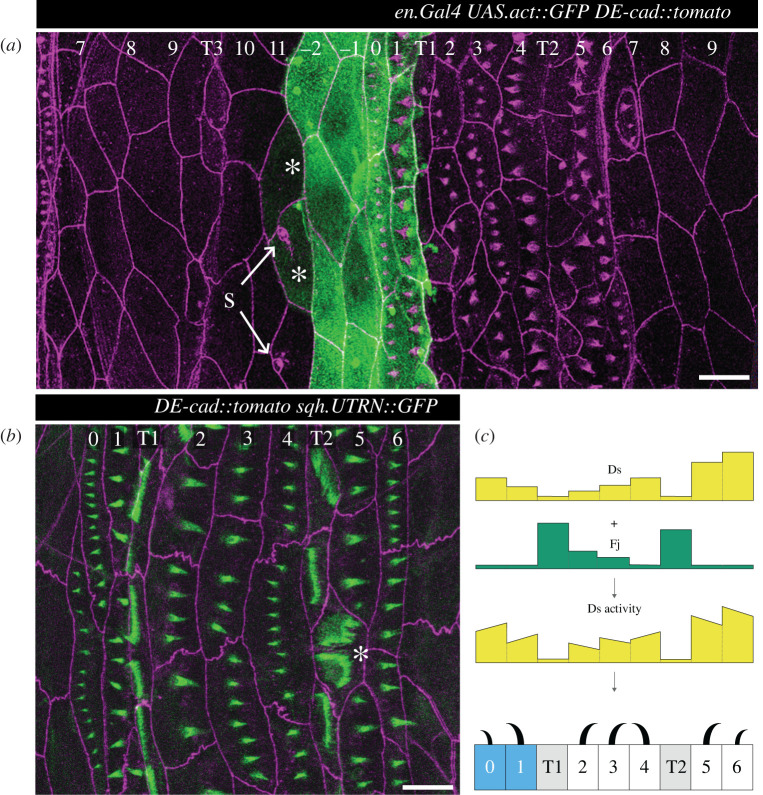
Larval ventral abdomen and Ds activity landscape. (*a*) Overview of a complete segment with cells expressing GFP under the control of the *engrailed* promoter, a marker of the P compartment [19,20]. Note that rows 7–11 and rows −2 and −1 indicate undenticulate rows of cells; before this paper the polarities of these cells were unknown, see later. GFP labels four rows of cells, between the most posterior row of the A compartment (identified by sensory cells, S) and the most anterior row of the following segment (tendon cells T1, see [18]). This driver occasionally also weakly labels a few cells at the rear of the A compartment (asterisks), but we have found that these cells do not express other P markers such as *hedgehog* (data not shown). Cell outlines and denticles are labelled in magenta (DE-cad::tomato). Arrows point to sensory cells (s) that we used as positional markers. (*b*) Ventral denticulate area of a mid-second-stage larva. Predenticles (rows 0 to 6) and tendon cells (rows T1 and T2) are marked in green (UTRN::GFP, labelling actin), and cell boundaries in magenta (DE-cad::tomato). The rows are not completely regular; here, one T2 cell contacts two row 6 cells at the posterior (asterisk)—typically, T2 only contacts row 5 cells. (*c*) A partially documented model of the landscape of Ds and Fj and therefore of PCP in the wild type [15,17]. In this model, a presumed low level of *ds* expression together with a documented high level of Fj reduces Ds activity in T1 and T2. The sloped line in each cell indicates different amounts of Ds activity at its anterior and posterior limits, the direction of the slope correlating with the cell's polarity. Denticle polarity is shown below and is a readout of the presumed landscape of Ds activity: each cell points its denticles towards the neighbour with the higher Ds activity. Two rows of the P compartment are highlighted in blue, tendon cells are shaded in grey. Anterior is to the left in all figures. Scale bars: 20 µm.

Each segment of the larva is divided by cell lineage into an anterior (A) and a posterior (P) compartment. In the adult abdomen, the A and P compartments are thought to be approximately coextensive with opposing gradients of Ds activity [16] and if such gradients were present in the larva then they could explain most of the denticle polarities. However, in the larva, in addition to the normal denticulate cells, there are three interspersed rows of muscle attachment cells [15,17,18] and our experiments suggest that two of these three rows have exceptionally low Ds activity which can affect the polarity of neighbouring cells (figure 1) [15,17]. At this point we are not clear how much the final pattern is determined by pervasive gradients of Ds activity or how much by these local effects of the muscle attachment cells plus propagation.

One outstanding difficulty in applying present models to the whole segment is that more than half the cells do not make denticles and their polarities are not known. In this paper we have solved that difficulty by measuring the molecular polarities of these uncharted cells in two complementary and different ways and this allows us to extend model-building to the entire segment. With the same purpose we have also measured the amount of Ds expression in each intercellular junction across the entire segment.

Depending on the pattern of Ds activity, individual cells will acquire different numbers of Ds-Ft and Ft-Ds heterodimers at opposite cell faces. Generally this difference will explain the polarity of the whole cell; however, sometimes, and depending on the disposition of neighbouring cells, two regions of a single cell can have opposing polarities [17]. To explain this phenomenon it has been argued that polarity of individual cells or parts of cells would depend on local ‘conduits’ that run between opposing cell faces to mediate their comparison. In this paper we reinvestigate these multipolar cells in an experimental situation.

There is some evidence that suggests that these conduits acting within the Ds/Ft system could be microtubules and might polarize the cell by orienting the intracellular transport of molecules and vesicles [21,22]. Indeed Harumoto *et al.* reported that, in one particular region of the pupal wing, the majority of microtubules are aligned near-parallel with the axis and direction of PCP (the direction of PCP is defined by the orientation of hairs) and, when growing, they show a small but statistically significant ‘bias’ in polarity [22]. By bias we mean a net difference between the number of microtubules growing within a particular angle interval and the number of microtubules growing 180 degrees away; for instance we might see more microtubules growing distally (i.e. in the same direction as the hairs) than in the opposite direction. Harumoto *et al.* therefore proposed that, in general, the Ds/Ft system controls the orientation of microtubules that would subsequently polarize cells by serving as oriented conduits in the polarized transport of PCP components [22]. Tests of this hypothesis in the adult abdomen have given mixed results [23–25]. Results from both wing and the abdomen are conflicting; regions of both appear to be polarized independently of the microtubules [25]. In the hope of clarifying this confusing situation we now report our studies of microtubule orientation *in vivo* in the larva. The larva has some advantages over imaginal discs or the adult abdomen: individually identifiable cells have a defined polarity and larval cells are much larger than the adult cells allowing more precision in plotting of the orientation of the microtubules. Several analyses of our own results on the larval abdomen and of raw data kindly provided by Axelrod from the pupal abdomen [24,25] do not support the hypothesis that PCP is oriented by microtubules.

In this paper we add to our knowledge of PCP in the larval segment; our two most important findings are to define cell polarity in all the cells of the entire segment and to provide data arguing strongly that orientation of the microtubules does not correlate with the axis of denticle polarity.

## 2. Materials and methods

### 2.1. Mutations and transgenes

Flies were reared at 25°C on standard food. The FlyBase [26] entries for the mutant alleles and transgenes used in this work are the following: *ds*: *ds^UA071^*; *en.Gal4*: *Scer\GAL4^en-e16E^*; *sr.Gal4*: *sr^md710^*; *UAS.act*::*GFP*: *Dmel\Act5C^UAS.GFP^*; *UAS.DsRed*: *Disc\RFP^UAS.cKa^*; *UAS.EB1*::*EGFP*: *Eb1^UAS.GFP^*; *UAS.ectoDs*: *ds^ecto.UAS^*; *UAS.LifeAct*::*mCherry*: *Scer\ABP140^UAS.mCherry^; UAS.RedStinger: Disc\RFP^DsRedT4.UAS.Tag:NLS(tra)^*; *UAS.ovo*: *ovo^svb.Scer\UAS^*; *act > stop > d*::*EGFP*: *d^FRT.Act5C.EGFP^*; *DE-cad*::*tomato*: *shg^KI.T:Disc\RFP-tdTomato^*; *ds::EGFP:* *Avic\GFP^ds-EGFP^*; *hs.FLP*: *Scer\FLP1^hs.PS^*; *sqh.UTRN*::*GFP*: *Hsap\UTRN^Scer\UAS.P\T.T:Avic\GFP-EGFP^*; *tub > stop > Gal4*: *Scer\GAL4^FRT.Rnor\Cd2.αTub84B^*.

### 2.2. Experimental genotypes

**Figure 2.**
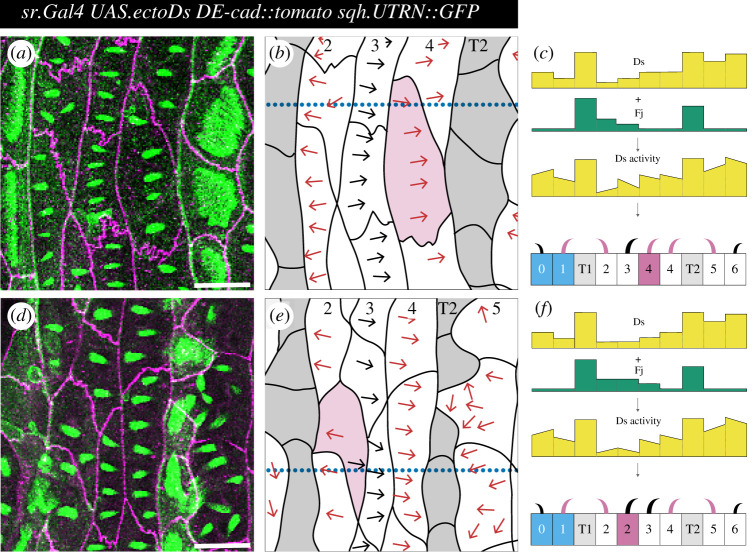
PCP and atypical cells in polarity-modified larvae. Denticulate areas of polarity-modified larvae: (*a*–*c*) an atypical cell in row 4 (having two posterior neighbours with different Ds activity), and (*d*–*f*) an atypical cell in row 2 (having two anterior neighbours with different Ds activity). Predenticles and denticles in rows 1, 2 and 4, 5 with polarity opposite from wild type are highlighted in magenta. (*a*,*d*) Images of predenticles, tendon cells and cell boundaries labelled as in figure 1*b*. (*b*,*e*) Schemes of cell outlines and predenticle orientation. (*c*,*f*) Models of polarity-modified larvae, Ds activity landscape and denticle polarity in cross sections taken at the dotted blue lines in *b*,*e*. Blue shading indicates P compartment cells, grey denotes tendon cells, magenta marks the atypical cell. Note that, contrary to wild type [17], in polarity-modified larvae row 4 atypical cells are monopolar (*a*,*b*), while row 2 atypical cells are multipolar (D,E). For quantitation of predenticle polarity in row 4 and row 2 atypical cells of wild-type and polarity-modified larvae, table 1. Scale bars: 20 µm.

(Figure 1*a*) *y w hs.FLP/ w; DE-cad::tomato/ en.Gal4 UAS.act::GFP*.

(Figure 1*b*) *w; DE-cad::tomato sqh.UTRN::GFP*.

(Figure 2, and table 1) *w; ds^UA071^ DE-cad::tomato sqh.UTRN::GFP/ DE-cad::tomato sqh.UTRN::GFP; sr.Gal4/ UAS.ectoDs*.

**Table 1.** Atypical cells: quantitation of predenticle polarities in relation to neighbouring cells, showing the effect of over expressing *ds* in the tendon cells.

wild type				*sr.Gal4 UAS.ectoDs*			
anterior neighbour	predenticle polarity of atypical row 2 cells^a^		posterior neighbour	anterior neighbour	predenticle polarity of atypical row 2 cells^c^		posterior neighbour
	anteriorly	posteriorly			anteriorly	posteriorly	
T1 cell	0	44^b^	row 3 cell	T1 cell	61	8^d^	row 3 cell
row 2 cell	0	52^b^	row 3 cell	row 2 cell	7^e^	49	row 3 cell

anterior neighbour	predenticle polarity of atypical row 4 cells^f^		posterior neighbour	anterior neighbour	predenticle polarity of atypical row 4 cells^h^		posterior neighbour
	anteriorly	posteriorly			anteriorly	posteriorly	
row 3 cell	207	0	T2 cell	row 3 cell	5	119^i^	T2 cell
row 3 cell	105^g^	45	row 4 cell	row 3 cell	0	99^i^	row 4 cell

^a^Predenticles of 39 atypical cells from 15 larvae. Fischer's exact test *p*-value = 1.

^b^8 predenticles with an unclear position were allocated equally to these classes.

^c^Predenticles of 42 atypical cells from 28 larvae. Fischer's exact test *p*-value <2.2^−16^.

^d^6 and

^e^3 predenticles with an unclear position were arbitrarily added to these classes.

^f^Predenticles of 74 atypical cells from 21 larvae. Fischer's exact test *p*-value < 2.2^−16^.

^g^18 predenticles with an unclear position were arbitrarily added to this class, in favour of the null hypothesis.

^h^Predenticles of 40 atypical cells from 20 larvae. Fischer's exact test *p*-value = 0.068.

^i^14 predenticles with an unclear position were allocated equally to these classes.

**Figure 3.**
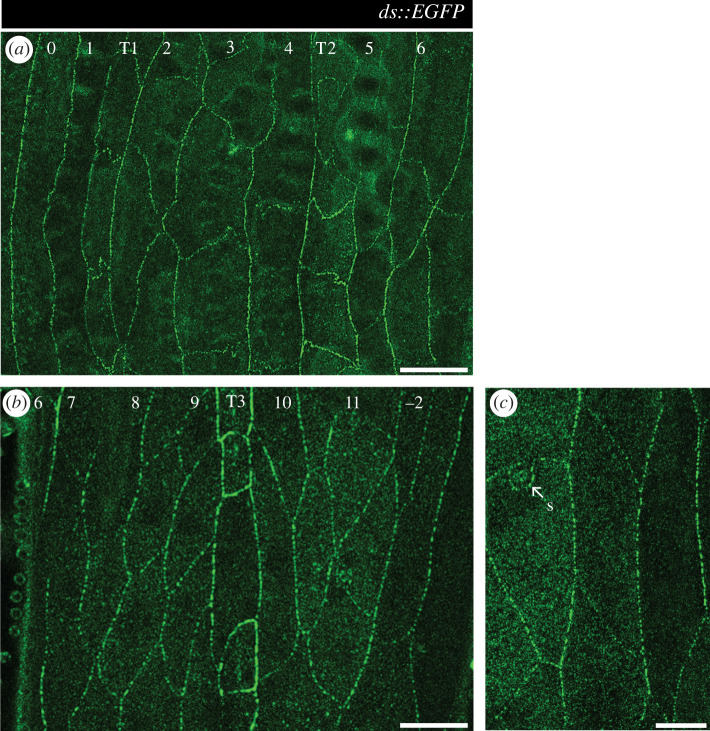
Ds localization in the larval ventral abdomen. Larvae expressing *ds*::EGFP from the tagged endogenous *ds* locus [14] show a ubiquitous punctate pattern of fluorescence that concentrates on plasma membranes. (*a*) Denticulate and (*b*) undenticulate areas of early-second-stage larvae; the cell rows exhibit no obvious differences in *ds* expression or distribution, with the exception of the strong signal around T3 tendon cells. (*c*) Detail of Ds localization in puncta at the cell membrane. 0 to 6, denticle cell rows. 7 to −2, undenticulate cell rows. S, sensory cell. T1, T2, T3, tendon cell rows. Scale bars: 20 µm (*a*,*b*), 10 µm (*c*).

**Figure 4.**
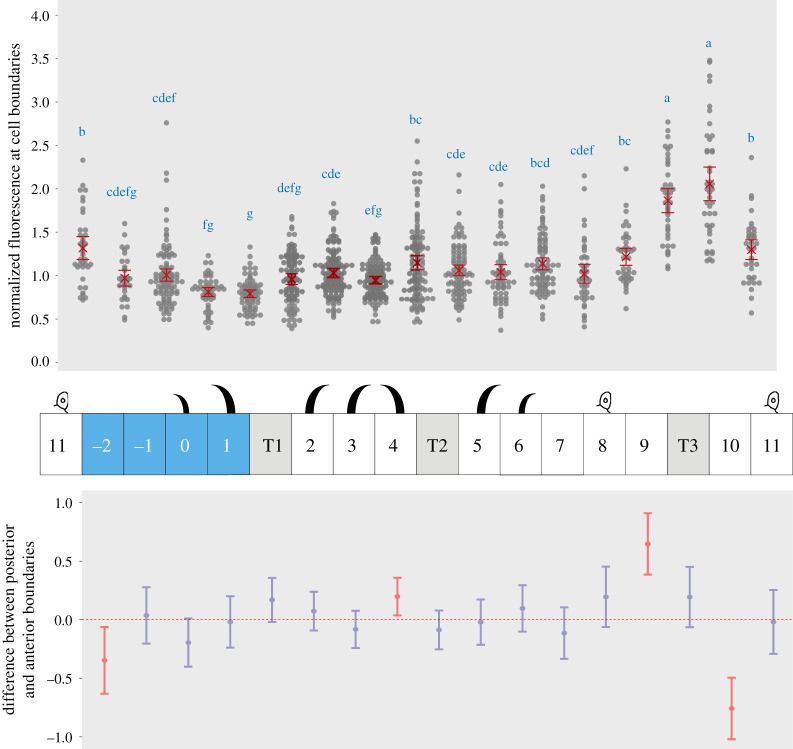
Quantitation of Ds levels at cellular interfaces across the segment. (Top) Dot plot of normalized fluorescence intensity maxima corresponding to amounts of Ds at boundaries between cell rows of the larval ventral abdomen. Data are pooled from 12 (denticulate area) and 5 (undenticulate area) images of different larvae. Mean value and 95% confidence interval for each interface are indicated in red. Letters arise from Tukey's multiple comparison test between all interfaces; in the Tukey's test, comparisons between pairs belonging to a group with the same letter show a *p*-value equal to or greater than 0.05. Groups can be assigned more than one letter, reflecting ‘overlap’ between different groups. The graph shows no evidence for a segment-wide gradient of Ds accumulation at the cell membranes, however the 9/T3 and T3/10 boundaries are significantly different from all others, indicating a clear peak anterior to the A/P boundary. (Middle) Diagram of denticle polarity, as in figure 1*c*. Sensory cells identify rows 8 and 11. (Bottom) Comparisons between Ds amounts at posterior and anterior interfaces of each cell row. Differences in mean normalized fluorescence at the opposite sides of a cell are calculated with 95% confidence interval by Tukey's test. Red indicates a significant difference. Note the significant and opposite differences in cell rows 9 and 10, highlighting the presence of a fluorescence peak around T3.

**Figure 5.**
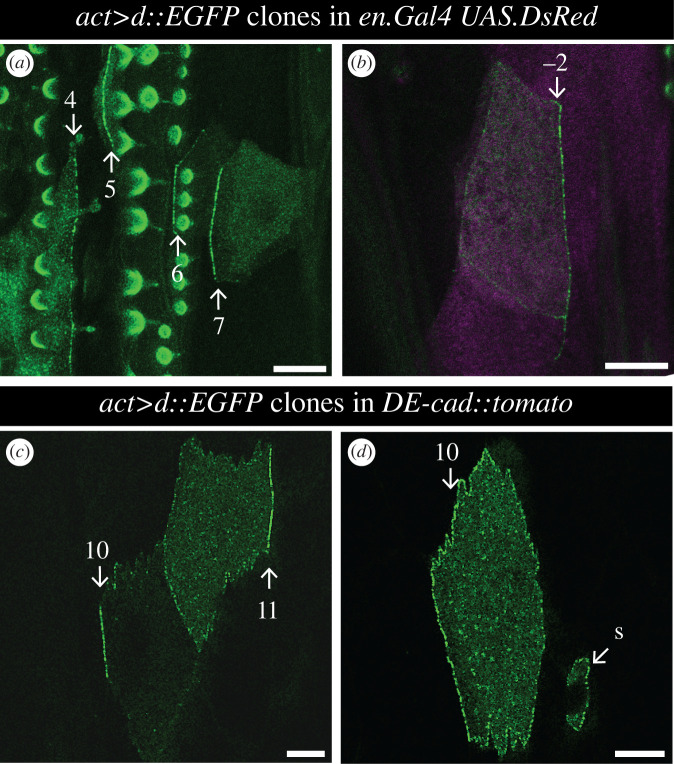
D polarity at the plasma membrane in small clones. (*a*) Several cells of the A compartment expressing *d::EGFP*: in row 4, where denticles point anteriorly, D is mostly on the posterior membrane; in rows 5, 6 and 7, with posterior-pointing polarity, D accumulates instead at the anterior face of the cells. Round or comma-like structures are due to autofluorescence from overlying denticles. (*b*) A posterior cell (row −2) accumulates D at its rear, arguing for anterior-pointing polarity. P compartment is labelled in magenta by *en.Gal4 UAS.DsRed*. (*c*) Cells of rows 10 and 11, where D localizes on the anterior and posterior sides of the plasma membrane, respectively (see electronic supplementary material, figure S2 for cell outlines). (*d*) Row 10 cell with more D on the anterior side of the cell membrane, suggesting its polarity points backwards. The sensory cell process associated with row 11 also expresses *d::EGFP*, and as with other cells from row 11 has most D at the posterior side. S, sensory cell. Scale bars: 10 µm.

**Figure 6.**
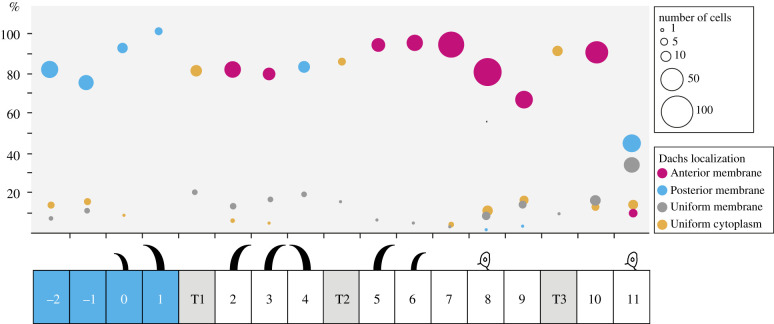
The localization of D cell by cell. D localization in all the cell rows, derived from the analysis of small clones expressing *d::EGFP*. Cells where D accumulates on just the anterior side of the plasma membrane contribute to red circles (anterior membrane), cells where D is only on the posterior side to blue circles (posterior membrane), cells where D is enriched at the plasma membrane but in an unpolarized manner to grey circles (uniform membrane), and cells where D is homogeneously distributed in the cytoplasm to orange circles (uniform cytoplasm). The position of each circle denotes the cell row and percentage of cells with the indicated D localization in that row; circle area is proportional to the number of cells represented. Since D is thought to accumulate on the side of a cell facing the neighbour with the least Ds, the pattern of D polarity in the undenticulate region suggests that there is a peak of Ds activity in row 10 (figure 9 for full model). *n* = 594 cells from a total of 44 larvae.

**Figure 7.**
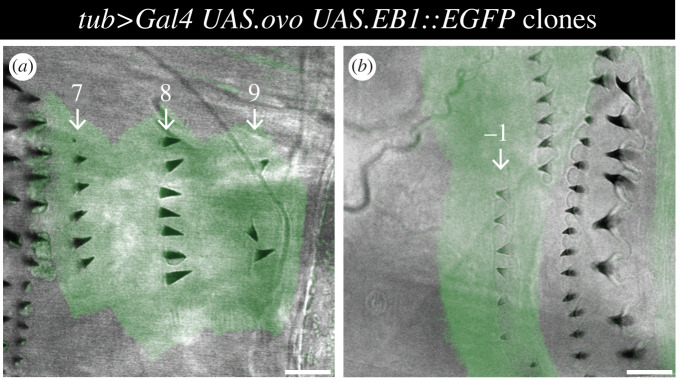
*ovo*-overexpressing clones in normally undenticulate areas of the epidermis. (*a*) Clone in the A compartment (cell rows 7, 8 and 9), marked with EGFP and producing ectopic denticles that point backwards. (*b*) Clone in the P compartment (cell row −1), ectopic denticles pointing forwards. Note that denticles are produced somewhat sporadically and that denticle numbers vary per cell. Scale bars: 10 µm.

**Figure 8.**
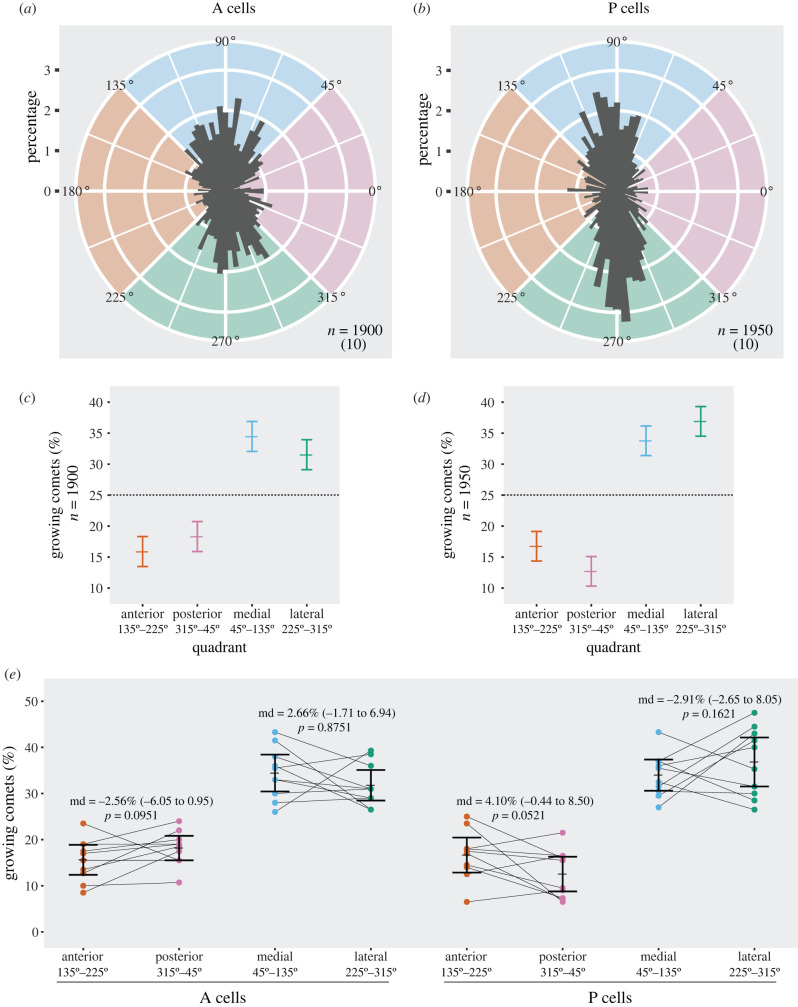
Analysis of microtubule polarity in larval epidermal cells. (*a*,*b*) Rose diagrams showing the distribution of growing microtubule direction in cells of the (*a*) anterior and (*b*) posterior compartment. EB1 comets are grouped in bins of 4 degrees, the length of each bin indicating the percentage of comets with a specific orientation. Comets pointing to the left (135–225 degrees, orange quadrant) grow anteriorly, comets pointing to the right (315–45 degrees, pink) posteriorly, up (45–135 degrees, blue) are medial, and down (225–315 degrees, green) are lateral; *n* is the total number of comets tracked, from the number of cells/larvae indicated in parenthesis. (*c*,*d*) Frequency of microtubules with either anterior, posterior, medial or lateral orientation in (*c*) A cells and (*d*) P cells. Comets are sorted into four sectors of 90 degrees centred on the anteroposterior and mediolateral axes. The 95% confidence interval for all comets in each quadrant is calculated according to Sison & Glaz [27]. (*e*) Dot plot comparing the orientation of microtubules within each cell of the A and P compartment. For every cell, the fraction of comets falling into the anterior quadrant is plotted next to the fraction in the posterior quadrant, medial next to lateral. Lines connecting the twin values from the same cell emphasize the high variability between individuals. Mean percentage and 95% confidence interval of the mean for each set of cells are shown. Overlying numbers display the exiguous difference between means (md) of the anterior versus posterior and medial versus lateral quadrants, with 95% confidence interval estimated by recalculating the difference of the means after resampling the data 10 000 times and finding the 0.025 and 0.975 quantiles of the resulting distribution of values; *p*-values were obtained as the frequency of resampled differences of the means that were greater than the observed.

(Figures 3 and 4) *w; ds::EGFP FRT40A*.

(Figures 5*a*,*b* and 6) *y w hs.FLP/ w; en.Gal4 UAS.DsRed/+; act > stop > d::EGFP/+*.

(Figures 5*c*,*d*, electronic supplementary material, figure S2, 6) *y w hs.FLP/ w; DE-cad::tomato; act > stop > d::EGFP/+*.

(Figure 7, electronic supplementary material, figure S3) *y w hs.FLP/w; tub > stop > Gal4/DE-cad::tomato; UAS.ovo/ UAS.EB1::EGFP*.

(Figure 8, electronic supplementary material, figures S4, S6, and movies 1, 2) *y w hs.FLP/w; tub > stop > Gal4/DE-cad::tomato; UAS.EB1::EGFP/UAS.LifeAct::mCherry*.

(electronic supplementary material, figure S1) *w; ds::EGFP FRT40A/+; UAS.ectoDs/sr.Gal4 UAS.RedStinger*.

### 2.3. Live imaging of larvae

To induce clones expressing *d::EGFP*, *ovo*, or *EB1::EGFP*, 2–4 h AEL embryos were heat shocked on agar plates with fresh yeast paste at 33°C for 30 min in a water bath. Larvae were grown at 25°C for 47–52 h and moved to fresh standard food for 2–4 h (tagged Ds, D, and EB1) or 10–15 hr (predenticles) before imaging. Second-stage larvae were washed in water and then immobilized between a glass slide and coverslip by exploiting the surface tension of a drop of Voltalef 10S oil or water. Epidermal cells in the A4–A7 abdominal segments of the larvae were imaged live through the cuticle using a Leica SP5 inverted confocal microscope with a 63×/1.4 oil immersion objective. Tagged fluorescent proteins were excited sequentially with 488 nm and 561 nm laser beams and detected with 510–540 nm and 580–630 nm emission filters, using Leica HyD hybrid detectors.

### 2.4. Quantification of Ds amounts at cellular interfaces

Ds::EGFP membrane distribution was analysed in the apical plane of ventral epidermal cells of early-second-stage larvae. Two juxtaposed areas of the segment (the denticulate and undenticulate regions) were imaged separately to grant sufficient resolution and subsequently merged, and maximum intensity projections of typically 4 µm stacks were used to compensate for ruggedness in the denticulate region. Between 3 and 12 images from different larvae were acquired and aligned to the mediolateral axis using rows of tendon cells as reference. Ten straight lines parallel to the anteroposterior axis and 4 µm wide were drawn over the images at random heights, and the profile of average fluorescence intensity along each line was plotted. Each profile displayed peaks where the line intersected cell boundaries: the fluorescence maxima were quantified using the BAR collection of ImageJ routines [28] and manually assigned to the respective cellular interfaces. Due to cell morphology and image noise not every line could provide a measure for each interface, therefore for every image a value of mean intensity was calculated only for cell boundaries intersected by at least 3 lines. The mean of means of all boundaries in an image was used as reference to normalize the fluorescence intensity maxima.

### 2.5. Mapping of D polarity

D polarity at the plasma membrane was assessed over the whole segment by analysing a total of 594 cells from small clones expressing *d::EGFP* in the ventral epidermis of 44 different larvae. Each cell was assigned a row number and polarity: rows of cells were identifiable by proximity to conspicuous landmarks like denticles, sensory cells and tendons with unique shape, while polarity was scored by eye based on whether D::EGFP fluorescence was exclusively on the anterior (Anterior membrane) or posterior (Posterior membrane) side of their plasma membrane, unpolarized but clearly enriched at the membrane (Uniform membrane), or homogeneously distributed in the cytoplasm (Uniform cytoplasm).

### 2.6. Analysis of microtubule growth direction

Orientation of growing microtubules was analysed following EB1::EGFP comets in ventral larval epidermal cells. Clonal expression of *EB1::EGFP* was necessary to avoid interference from the strong signal of underlying muscle cells, and undenticulate regions were preferred because denticles obscured the fluorescent signal. Early-second-stage larvae were mounted in a small drop of water ensuring their posterior spiracles were out of the liquid, and movies of individual cells were recorded at 5.16 s intervals for typically 5 min, imaging a single 0.773 µm apical confocal plane. Movie frames were registered using the ImageJ plugin Stackreg [29] to account for slight movements of the larvae. Cells were then aligned to the mediolateral axis using the T3 row of tendon cells and rows of denticles as references, and cells situated in the right hemisegments were flipped to match the mediolateral orientation of the left hemisegment cells. Two cells, one in the A compartment (row 7 or 8) and one in the P (row −2 or −1), were selected from each of 10 larvae and pooled for blind analysis. Comets were traced manually using the ImageJ plugin MtrackJ [30], sampling all the visible comets within each cell for as many time points as were necessary to count 150–200 comets per cell, and angles of the comets' trajectories relative to the anteroposterior axis of the larva were derived from the first and last time point of their tracks.

### 2.7. Data analysis

Data analysis was carried out in R 3.5.3 [31], using the *CircMLE* [32], *circular* [33], *DescTools* [34], *dplyr* [35], *ggplot2* [36] and *mosaic* [37] packages.

## 3. Results

### 3.1. Comparing wild-type and polarity-modified larvae

#### 3.1.1. Background

In this section we reexamine and test the model as exemplified by those single cells described as ‘atypical’ in which one face of the cell's membrane abuts two different neighbours [17]. Some of these cells are multipolar and these exemplify very strongly the argument that PCP stems from a comparison between the facing membranes of a single cell. These atypical and multipolar cells are now studied in ‘polarity-modified’ larvae, in which the overall segmental polarity has been considerably modified by experiment. Unlike previously, we study the predenticles, that is denticles observed prior to the deposition of cuticle.

We compare the cell polarity of wild-type [15,17] and polarity-modified larvae (figure 2). To make the polarity-modified larvae, we engineer increased expression of an active form of *ds* in T1 and T2 cells (*sr.Gal4 UAS.ectoDs* [15]); this changes the landscape of Ds activity, making peaks (instead of troughs, as in the wild type) in T1 and T2. Consequently, the polarities of rows of cells 1, 2, 4 and 5, that abut T1 and T2, now point inwards; that is reversed from the wild type (figure 2). The other rows, 0, 3 and 6 could also be affected because polarity can be propagated beyond the neighbouring cells [8,9,15]. To explain further how the Ds/Ft machine propagates polarity changes from cell to cell: an increase in Ds activity in cell *a* attracts more Ft on the facing membrane of cell *b*. On that facing membrane more Ft tends to exclude Ds activity, enabling more Ds to accumulate on the far side of cell *b* which will, in turn, draw more Ft to the facing membrane of cell *c* [5,7].

#### 3.1.2. Atypical cells

In all larvae, the numbered cell rows are often irregular and some atypical cells may individually abut on the same side two neighbours, each with a different level of Ds activity. We compare the predenticles of atypical cells in wild-type and polarity-modified larvae. In the wild type, one posterior part of cell *a* in row 4 may contact a T2 neighbour with a lower Ds activity than row 3 (the associated predenticles in this region of cell *a* point anteriorly) and a separate part of cell *a* may contact a row 4 neighbour with a higher Ds activity than row 3 [17]. However, in the polarity-modified larvae, the predenticles of nearly all cells of row 4 (typical and atypical cells) point posteriorly—this is as expected from the model because *both* types of posterior neighbour that can abut a row 4 cell (T2 and another row 4 cell) now have higher levels of Ds activity than the anterior neighbour, a row 3 cell (figure 2*a–c* and table 1). However for these polarity-modified larvae, some single atypical cells of row 2 have two anterior neighbours—cells of T1 and row 2—that are higher and lower in Ds activity than the posterior neighbour of the atypical cell, respectively. Consequently, the model predicts that their associated predenticles should point forwards in that part of the cell that abuts T1 and backwards in that part of the same cell abutting row 2, and they do (figure 2*d*–*f* and table 1). There are some quantitative differences between the current data and the wild types we scored earlier [17] (see legend to table 1). Nevertheless, these results, especially on the polarity-modified larvae, confirm and strengthen a model of PCP in which cells in a tissue are polarized due to an underlying gradient of Ds activity. They are not sufficient to exclude a model in which polarization depends only on local interactions between cells.

### 3.2. Direct assessment of Ds distribution in both wild-type and polarity-modified larvae

We measure the native Ds distribution using a tagged Ds molecule expressed as in the wild type. Ds accumulates as puncta in the membrane (figure 3) [14,38] and, presumably, the puncta contain or consist of Ds-Ft heterodimers [39].

We previously inferred but did not show directly a supracellular gradient in Ds activity that rises within the A compartment reaching a peak near the rear of that compartment and then falling into the P [16]. We therefore quantified and compared the amount of Ds localized at cell junctions in all rows of the segment in the larval ventral epidermis. These measurements do not evidence an overall gradient. However, both junctions 9/T3 and T3/10 show a higher amount of tagged Ds than the other boundaries; these junctions are located near the rear of the A compartment (figure 4). We applied the same quantitation technique to polarity-modified larvae and found that the distribution of Ds is altered from the wild type as expected (electronic supplementary material, figure S1), in a way that validates our quantification technique and consequently the existence of a peak of Ds levels near the rear of the A compartment in the wild type (figure 4).

### 3.3. The location of Dachs

The myosin-related molecule D is a marker of polarity and localized by the Ds/Ft system [5,14,40–42]. It is usually asymmetrically distributed on a polarized cell and is thought to co-localize with the face of the cell associated with the most Ds [14,41,42]. We map D to the membranes of individual cells in the larval epidermis by making small clones of cells that express tagged D; this allows the distribution of D on a particular cell to be assessed so long as the neighbour(s) does not contain any tagged D.

**Figure 9.**
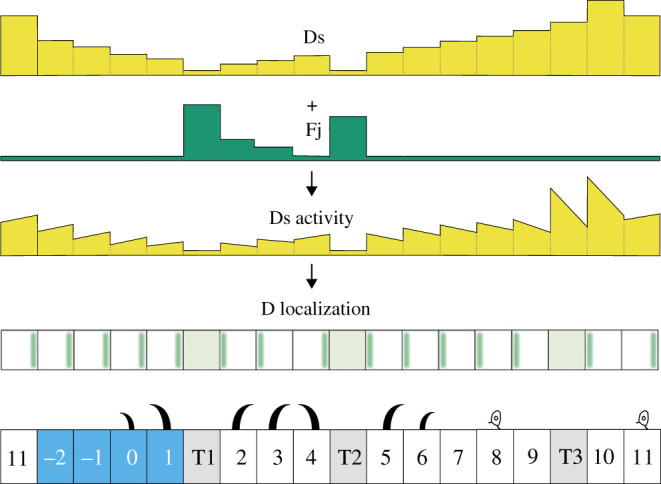
Model of Ds activity and planar cell polarity in the larval ventral epidermis. The strong Ds accumulation on both sides of T3 tendon cells (figures 3 and 4) suggests that *ds* expression is high in T3 itself and/or its neighbours. In addition, D::EGFP clones (figures 5 and 6) and ectopic denticles (electronic supplementary material, figure S3A) show that polarity of row 10 points backwards, away from T3, implying that Ds activity is higher in row 11 than in T3. These two observations combined argue that *ds* expression peaks in row 10, two cells anterior to the A/P border, with Ds activity also high in T3 and row 11. Graded *ds* expression forwards and backwards from this peak together with high levels of *fj* expression in tendon cells determine the landscape of Ds activity, now extended to the undenticulate region. The Ds gradient indicated has not been confirmed, it is a speculation. Our data suggest that if there is a pervasive gradient, it will be shallow, perhaps even more shallow than shown. The differences in Ds activity between each cell's anterior and posterior sides orient D accumulation; D localizes to the side that has the highest Ds activity and ‘sees’ the lowest Ds activity in its neighbour. D asymmetrical distribution precisely matches the pattern of cell polarity revealed by denticles, as demonstrated by direct visualization of tagged D in the whole segment and induction of denticles in normally naked cells. Cell 11 is shown with some ambiguity, because that is what we find (see main text). Blue shading indicates P compartment cells, grey shading, tendons.

We examine the distribution of D in wild-type larvae in order to reveal the molecular polarity of cells that lack denticles (figures 5 and 6). In the P compartment, all the denticulate and undenticulate cells show a consistent molecular polarity, D being localized posteriorly in the cell. Most cells of the A compartment have the opposite polarity, with D located anteriorly. In both compartments, the location of D in the denticulate cells correlates in all cases with the denticle polarity, and this includes the cells of rows 0, 1 and 4 whose denticles point forward. The tendon cells, T1, T2 and T3 can express D but it is mostly cytoplasmic in location. The cells flanking T1 and T2 (but not T3) accumulate D at the membrane abutting the tendon cells. Unlike all the other rows, cells of row 11 show some variation in the localization of D: about 45% localize it at the posterior cell membrane, as do cells in the P compartment; in 35% it is at the membrane but not asymmetrically localized and, in the remaining cells, D is either at the anterior or found only in the cytoplasm (figure 6). This means that the line where polarity changes from the A-mode to the P-mode is not at the A/P border [16] but anterior to it; suggesting that the second cell row anterior to the A/P cellular interface (row 10) contains the peak level of Ds activity. From that row, effects on polarity spread forwards into the A compartment and backwards into row 11 and the P compartment (see model in figure 9).

The localization of D is not always continuous along the entire face of a cell. When the plasma membrane of one side of an atypical cell A abuts two separate cells, and our model implies that these two cells have different levels of Ds activity, then the D from cell A is localized at the interface with just one of those cells, on that part of the membrane that has most Ds activity (cells 10 and 11 in figure 5*c*, and electronic supplementary material, figure S2, see legend). This suggests that different parts of a single cell's membrane can compete for D.

### 3.4. *ovo*-expressing clones reveal otherwise unseen polarity

Small clones that overexpress *ovo* in naked areas often produce denticles in embryos [43,44]. We made marked clones in larvae and these also generally made denticles. The denticles showed a consistent orientation, pointing forwards in P and backwards in most of A, exactly mirroring the polarity pattern as identified by D localization (figure 7*,* compare with figure 6). Thus, cells of row 11 at the rear of the A compartment mostly made denticles that pointed forwards (figure 7) as is characteristic of cells belonging to the P compartment. Just as signalled by the localization of D, in a minority of row 11 cells, polarity was ambiguous with denticles pointing in various directions (electronic supplementary material, figure S3). The denticles belonging to the cell row 10 anterior to row 11 always pointed backwards and denticles of the row behind row 11 (row −2 of the P compartment) always pointed forwards.

### 3.5. Does the orientation of growing microtubules correlate with PCP?

We study the orientation of growing microtubules (using EB1 comets [45,46]) in the large epidermal cells of the ventral larva. Our main data are collected from identified A cells of rows 7–8 (direction of PCP is posterior) and identified P cells of rows −2 and −1 (direction of PCP is anterior; figure 6); the classification of the A and P cells as having opposite polarities is based on studies of the larval ventral abdomen described above. To assess the orientation of growing microtubules, we took 10 larvae, made films and studied one A and one P cell from each (electronic supplementary material, movies 1, 2). The growing microtubules were then recorded vis-à-vis the axis of the larva by one person (S.P.) who was blinded to the identity of each of the 20 cells he was scoring. The orientations of about 4000 EB1 comets are shown and analysed in figure 8.

In the wing, the predominant alignment of the microtubules is close to the axis of PCP [22,47]. By contrast, in the larval epidermal cells, in both A and P compartments, the majority of the microtubules are aligned perpendicular to the anteroposterior axis, the axis of PCP (figure 8*a,b*). To analyse our data and following the approach in the wing, the comets of the larvae are sorted into four 90 degree quadrants centred on the anteroposterior and mediolateral axes and their frequencies plotted. The quadrants are described as ‘anterior’, ‘posterior’, ‘medial’ and ‘lateral’ (figure 8*c*,*d*). The axis of PCP lies in the anteroposterior axis, but in A compartment cells, 66% of the total angles of growth fall within the medial and lateral sectors, while in the P compartment the comparable figure is 71%. Clearly there is no overall correlation between microtubular orientation and PCP, belying the hypothesis that microtubular orientation is causal for PCP.

However, we could look for a limited correlation between the orientation of growing microtubules and the direction of PCP. For example, considering only the minority of microtubules within the anterior and posterior sectors, we find insignificant differences in polarity (figure 8*c*,*d*). In A cells the proportion of all microtubules that grow anteriorly is 15.8% with a 95% CI of [13.5 to 18.2] and the proportion that grow posteriorly is 18.3% [15.9 to 20.6]. In P cells it is the reverse; 16.7% grow anteriorly [14.4 to 19.1] and the proportion that grow posteriorly 12.7% [10.3 to 15.0]. There was a comparably weak bias in the medial and lateral quadrants: in A cells a larger proportion of all microtubules grow medially 34.4%[32.0 to 36.8] than laterally 31.5% [29.1 to 33.8] while the reverse bias occurs in P cells where more microtubules grow laterally 36.9% [34.5 to 39.2] than medially 33.7% [31.4 to 36.1] (figure 8*c*,*d*).

How uniform are the individual cells? To answer we group all the growing microtubules according to which cell (and larva) they come from and according to which of four 90 degree quadrants they fall into (figure 8*e*). Remarkably, in all sets, individual cells differ wildly from each other. Comparing the anterior versus posterior and medial versus lateral quadrants we find no strong evidence for a bias in the directions in which the microtubules grow—apart from the obvious and main finding that most of the microtubules grow more or less perpendicular to the axis of PCP.

Could there be a special subset of oriented microtubules perhaps aligned close to the anteroposterior axis, the axis of PCP, that might show a polarity bias that related to some function in planar polarity? There is no independent evidence favouring such a perspective. Nevertheless, to check we scan through the entire circumference in 22.5 degree sectors, measuring the amount of bias in the microtubules that fall within opposite pairs of sectors. There is no increase in bias in the sectors that included the axis of PCP in either the A or the P compartments, nor in nearby sectors. However, there is a local peak of bias within the A compartment: there is a significant bias in the number of growing microtubules within one pair of 22.5 degree sectors that is far away from the axis of PCP. Within the P compartment a similar peak of bias is centred near the mediolateral axis within two facing 22.5 degree sectors (electronic supplementary material, figure S4). But note that these biases represent only 2–3% of the total population of microtubules. Thus, although we found some irregularities in the circular distribution of growing microtubules, we find no correlation with the axis of PCP.

Axelrod's group kindly made their raw data from the pupal abdomen available to us and we treat them exactly as our larval data. Axelrod and colleagues grouped the angles of growing pupal comets into two unequal sets (two broad sectors of 170 degrees, each including the anteroposterior axis, were compared to each other, while the remaining microtubules were grouped into two narrow mediolateral sectors of 10 degrees each [24,25]). But for our analysis, to conform with how data on the wing have been presented [22,24,25], and to allow a comparison with our results, we subdivided their data into four 90 degree quadrants. Even more so than in the larva, the majority of the pupal microtubules are oriented orthogonally to the axis of PCP (electronic supplementary material, figure S5A–D): 69% of the total population of growing microtubules in the A compartment are aligned within the quadrants centred on the mediolateral axis, while in the P compartment the comparable figure is 73% (electronic supplementary material, figure S5C,D). This finding does not fit comfortably with a hypothesis that microtubular orientation drives PCP.

Further comparison of the Axelrod group's data on the pupa with ours on the larva show some quantitative differences. Unlike ours on the larva, their pupal data show statistically significant biases in the orientation of comets (electronic supplementary material, figure S5C,D). In A cells the proportion of all microtubules that grow anteriorly is 12.7% with a 95% CI of [11.3 to 14.1], significantly smaller than the proportion that grow posteriorly: 18.1% [16.6 to 19.5]. In P cells we see a reverse bias: 15.8% [13.3 to 18.2] grow anteriorly and 11.5% [9.1 to 13.9] posteriorly. Notably, there is a comparable and also significant bias in the medial and lateral quadrants but in the same direction in both compartments. In A cells a larger proportion of all microtubules grow laterally 38.1% [36.7 to 39.6] than medially 31.1% [29.7–32.5], and a similar bias occurs in P cells where 39.8% [37.4–42.3] grow laterally and 32.9% [30.5–35.3] grow medially (electronic supplementary material, figure S5C,D).

We then plotted all the growing microtubules according to which pupa they came from and according to which of four 90 degree sectors they fell into (electronic supplementary material, figure S5E). Individual pupae differ wildly from each other. In both our results on the larva and Axelrod's results in the pupa, there is considerable inconsistency between individuals (compare figure 8*e* with electronic supplementary material, figure S5E). Only when all cells are taken together is there any overall and significant polarity bias in Axelrod's data.

We classified the growing microtubules in Axelrod's data into 22.5 degree sectors and looked for an orientation bias within opposite pairs of sectors. We find examples of significant bias shown by the microtubules in various sector pairs and these are mostly not near the axis of PCP. In A cells there is a statistically significant and local peak of bias roughly 60–80 degrees divergent from the axis of PCP. In P cells there is a statistically significant and local peak of bias roughly 35–55 degrees divergent from the axis of PCP (electronic supplementary material, figure S4). These observations do not fit with the conjecture that a special set of oriented microtubules, in or close to the PCP axis, might be driving planar polarity.

Dividing the data into sectors gives the impression of biases in the anteroposterior as well as in the mediolateral axes (although these are non significant in the case of the larva). But, because we suspect that subdividing the angles into sectors may lead to erroneous conclusions we investigated the distributions of the angles as a whole. We took the angular data of the A and P cells of the larva and pupal abdomen and using a maximum likelihood model approach [32], we found that the best fit in all four cases is to a distribution with two peaks each roughly 90 degrees divergent from the axis of PCP (electronic supplementary material, figure S6). Unexpectedly, there are slight deviations of these peaks in the bimodal distributions; in all four distributions one of the peaks deviates 10 degrees from the mediolateral axis. Interestingly, the direction of deviation is opposite in the A cells to that in the P cells; in both sets of A cells one of the peaks is tilted 10 degree towards the posterior hemi-circumference, whereas in both sets of P cells one of the peaks is tilted 10 degrees towards the anterior hemi-circumference (electronic supplementary material, figure S6, see legend). These opposite deviations in A and P cells may be the basis of the apparent but weak biases we observe when dividing the data into four quadrants.

## 4. Discussion

### 4.1. A gradient model?

In trying to understand planar cell polarity, *Drosophila* has proved the most amenable and useful experimental system. Using the *Drosophila* larva, we have built a model of how the Ds/Ft system determines the pattern of polarity in the abdominal segment [16,17]. In this model the Ds/Ft system converts graded slopes in the expression levels of *ds* and *fj* into local intercellular differences in the levels of Ds activity, and into PCP without any intervention by the Stan/Fz system [5].

Here we have reexamined the model and extended it to those uncharted parts of the larval segment that lack denticles (figure 9). All the observations we have made give results that are consistent with and support the model. However it is not clear whether the model requires interactions between Ds, Ft and Fj to produce a multicellular gradient of Ds levels at the cell membranes, and expectations on this differ [39]. We originally proposed that the levels of Ds activity would be graded in opposite ways in the A and the P compartment and ultimately these gradients would be read out as PCP in each of the cells [16]. We imagined that multicellular gradients of Ds activity would persist and span the whole field of cells and this has been assumed by most [5,7,48,49] and actually detected, locally, in the migrating larval epidermal cells in the pupa [50]. Alternatively, once the arrow of polarity has been established in each cell, a feedback mechanism could result in a redistribution of bridges so that, ultimately, each cell would contain the same number of bridges, similarly disposed—there would be no persistent multicellular gradient in Ds activity (e.g. [39]). However there would still be differences in the dispositions and orientations of Ds-Ft bridges between the opposite membranes of each cell. Our current measurements of Ds levels do not settle the matter: we did not detect a pervasive gradient of Ds, but amounts were not flat either. We found a peak in Ds level located near the rear of the A compartment near where a Ds activity gradient was predicted to summit. However, a shallow Ds gradient could still exist—it might be missed because we quantify only the total Ds present in abutting pairs of membranes. This shortcoming means that the results can neither tell us the cellular provenance of the Ds we measure, nor reveal how much of it is in Ds-Ft or in Ft-Ds bridges within the apposed membranes. Thus, if any cell has a higher level of Ds, this Ds will bind more Ft in the abutting cell membrane, and, we believe, tend to exclude Ds from that abutting membrane. These effects will tend to even out the amounts of Ds in joint membranes and therefore tend to disguise any gradients, local peaks or troughs.

Could one build the segmental pattern of polarity using only a peak plus propagation, thereby managing without any initial gradient of *ds* expression? If so, a localized peak in amount of Ds at the rear of the A compartment (with a maximum in row 10) could affect polarity forwards into row 9 and beyond, and propagate backwards through row 11 into the P compartment. The single cell troughs in Ds activity in T1 and T2 would orient the polarity of the flanking cells to point away from these tendon cells. All these polarity effects would reinforce each other to make a more robust pattern. However, if there were no initial gradient of *ds* expression, only row 3 would present a problem; in order to explain why it points backwards, the trough of T1 in Ds activity would need to be deeper than that of T2 (fig. 4 in [15]). Perhaps it will prove important to note that the gradient model and the alternative localized peak and troughs model just outlined are not mutually exclusive and each can contain aspects of the truth.

Originally predicted to be at the A/P compartment border [16] we conclude now that a Ds peak occurs two cells anterior to that border, in row 10 (figure 9; a similar peak two cells from the A/P border has been described in the dorsal abdomen of the pupa [50]). This observation is supported by both D localization and the orientation of ectopic denticles formed by *ovo*-expressing clones. There are interesting implications: the peak in Ds protein at the cell junctions is in a cell that is flanked on both sides by A compartment cells, the most posterior of which (row 11) has ‘P type’ polarity. Why is this summit out of register with the lineage compartments? It could be that this peak is specified by a signal emanating from one compartment and crossing over to affect the next compartment. There are precedents for this kind of transgression [19,51–54]. Also, in the abdomen of the developing adult fly, Hedgehog signal spreads from the P compartment across into the A compartment and induces different types of cuticle at different distances [55].

Our results can best be interpreted, as others have done [14,40,56], as showing that D acts as an eloquent marker of a cell's polarity, is localized on the membrane with the most Ds and acts immediately downstream of the Ds/Ft system.

### 4.2. Microtubules and PCP

We have suggested [17] that intracellular conduits might be involved in a local comparison between facing membranes of a cell and shown here that this perspective successfully predicts which cells should become bipolar even in polarity-modified larvae. But there is still no direct evidence for the conduits, and no knowledge, if they do exist, of what they are. One could imagine a set of microtubules, initiated on the membrane, that could align more or less with the anteroposterior axis and traverse the cell to meet the membrane opposite. Indeed, Uemura's group [21] have proposed that microtubules, oriented by the Ds/Ft system, translocate vesicles carrying PCP components such as Frizzled (Fz) and Dishevelled (Dsh) to one side of a cell to polarize it. Their hypothesis began with observations on microtubule-dependent transport of tagged proteins *in vivo* in cells of the wing disc [21] and was extended by the use of EB1 comets to plot microtubule polarity in the pupal wing [22–25]. Harumoto and colleagues [22] studied the proximal part of the wing where they found a transient correlation, with a small majority of the microtubules growing distally, but there was no such correlation in the distal wing. Also, in *ds^–^* wings, distal regions show consistently polarized microtubules (a small majority now grow proximally), although the hairs in that region still point distally [22]. Likewise, while some studies of the adult abdomen demonstrate a local correlation between cell polarity and the orientation of limited subsets of microtubules, PCP in other parts did not show this correlation and the authors concluded that, in those parts, polarity is determined independently of the microtubules [25]. We have tested the hypothesis that microtubular orientation drives PCP in the larval abdomen of *Drosophila* and there it also meets serious difficulties. The greatest of these is that most of the microtubules are aligned orthogonally to the axis of PCP (this fact is also extractable from the pupal data kindly provided by Axelrod's group). Of the roughly 30% of all microtubules that fall into the two quadrants centred on the axis of PCP, there is a small net excess, corresponding to about 5% of the total, that could perhaps result in a net transport of vesicles in the direction of PCP. But even if this were so, more than 80% of the vesicles carrying cargo should arrive in the wrong part of the cell membrane.

Why are there apparent biases in microtubule orientation in the data? An analysis of the circular distribution of comets showed, in all the sets of data (ours and those of Axelrod's group), a deviation of 10 degrees in one of the peaks of the bimodal distribution of the angles (electronic supplementary material, figure S6). This deviation, plus the precise orientation of the 90 degree quadrants, may explain the apparent bias of microtubular orientation seen clearly in the Axelrod data and hinted at much more weakly in our data. How? Imagine a circular bimodal distribution composed of two separate unimodal distributions: the tails of both probability distributions would be closer and overlap more if the distance between the mean angles were reduced. In our cases, one of the tails of the distributions whose mean angles deviate by 10 degrees will decrease slightly the frequency of angles within one of the anteroposterior quadrants and concomitantly the other tail increase the frequency in the opposite anteroposterior quadrant. This deviation may have its origin in a correlation between cell shape and microtubular orientation [47,57,58] and in different cell shapes in the A and P cells; these are more obvious at or close to the A/P border [59].

The hypothesis of Uemura's group [21], which proposes that microtubules transport Fz to one side of the cell to polarize it, meets an additional problem in the larval abdomen. The normal orientations of the denticles in the larva does not require input from the Stan/Fz system; indeed the Ds/Ft system appears to act alone [7–9]. But could oriented microtubules be involved in PCP, even without any role of the Stan/Fz system? Our results from the larval abdomen say no. We cannot exclude the possibility of a small subset of stable microtubules (undetectable because they would not bind EB1), aligned with the anteroposterior axis and strongly biased in polarity, in the pupal or larval abdomens (or proximodistal axis in the wing). There is no evidence for such microtubules, but if they exist their number and bias in orientation must be strong enough to overcome the moving of vesicles on the unbiased dynamic microtubules we have studied.

## 5. Conclusion

We have enhanced our present model of how the Ds/Ft system generates the intricate polarity of the larval segment. The key element of this model is that each cell compares its neighbours and is polarized (and points its denticles) towards the cell presenting the most Ds activity. This hypothesis gains more support from our new results on the multipolarity of single cells. But we have not found out how the comparison is made: an attractive hypothesis by others was that oriented microtubules are the critical agent, but, if we interrogate our data for biases in polarity within all the growing microtubules, or if we select subsets of microtubules whose orientations are related to the axis of PCP, we do not find evidence for a link between microtubular polarity and the polarity of the denticles (the ‘direction’ of PCP). Using two different methods we demonstrated that undeticulated cells are also polarized and their polarity is as the model predicts, and that the point where the amount of Ds is, presumably, highest and from where, like a watershed divide, polarity diverges, is two cells away from the compartment border. We looked to demonstrate the predicted multicellular gradient of Ds but, possibly because of an insufficiency in our methods, we only found a localized peak (at the rear of the A compartment as the model requires). Thus, if there is a multicellular gradient of Ds activity, it must be very shallow. There's still much to do; still so much to learn.

## Supplementary Material

Supplementary figures

## Supplementary Material

Movie S1

## Supplementary Material

Movie S2

## Data accessibility

Data used in figures 4 and 8, electronic supplementary material, figure S1, S4–6 can be obtained from the University of Cambridge Open Access repository (https://doi.org/10.17863/CAM.53667).

## References

[1] Goodrich LV, Strutt D 2011 Principles of planar polarity in animal development. Development 138, 1877–1892. (10.1242/dev.054080) 21521735 PMC3082295

[2] Henderson DJ, Long DA, Dean CH 2018 Planar cell polarity in organ formation. Curr. Opin. Cell Biol. 55, 96–103. (10.1016/j.ceb.2018.06.011) 30015152

[3] Devenport D 2016 Tissue morphodynamics: translating planar polarity cues into polarized cell behaviors. Semin. Cell Dev. Biol. 55, 99–110. (10.1016/j.semcdb.2016.03.012) 26994528 PMC5005802

[4] Butler MT, Wallingford JB 2017 Planar cell polarity in development and disease. Nat. Rev. Mol. Cell Biol. 18, 375–388. (10.1038/nrm.2017.11) 28293032 PMC5826606

[5] Lawrence PA, Casal J 2018 Planar cell polarity: two genetic systems use one mechanism to read gradients. Development 145, dev168229 (10.1242/dev.168229) 30530515

[6] Carvajal-Gonzalez JM, Mlodzik M 2014 Mechanisms of planar cell polarity establishment in *Drosophila*. F1000Prime Rep. 6, 98 (10.12703/P6-98) 25580252 PMC4229721

[7] Casal J, Lawrence PA, Struhl G 2006 Two separate molecular systems, Dachsous/Fat and Starry night/Frizzled, act independently to confer planar cell polarity. Development 133, 4561–4572. (10.1242/dev.02641) 17075008 PMC2747022

[8] Donoughe S, DiNardo S 2011 dachsous and frizzled contribute separately to planar polarity in the *Drosophila* ventral epidermis. Development 138, 2751–2759. (10.1242/dev.063024) 21613320 PMC3109600

[9] Repiso A, Saavedra P, Casal J, Lawrence PA 2010 Planar cell polarity: the orientation of larval denticles in *Drosophila* appears to depend on gradients of Dachsous and Fat. Development 137, 3411–3415. (10.1242/dev.047126) 20826534 PMC2947754

[10] Simon MA, Xu A, Ishikawa HO, Irvine KD 2010 Modulation of Fat:Dachsous binding by the cadherin domain kinase Four-jointed. Curr. Biol. 20, 811–817. (10.1016/j.cub.2010.04.016) 20434335 PMC2884055

[11] Ishikawa HO, Takeuchi H, Haltiwanger RS, Irvine KD 2008 Four-jointed is a Golgi kinase that phosphorylates a subset of cadherin domains. Science 321, 401–404. (10.1126/science.1158159) 18635802 PMC2562711

[12] Brittle A, Repiso A, Casal J, Lawrence PA, Strutt D 2010 Four-jointed modulates growth and planar polarity by reducing the affinity of Dachsous for Fat. Curr. Biol. 20, 803–810. (10.1016/j.cub.2010.03.056) 20434337 PMC2958304

[13] Ambegaonkar AA, Pan G, Mani M, Feng Y, Irvine KD 2012 Propagation of Dachsous-Fat planar cell polarity. Curr. Biol. 22, 1302–1308. (10.1016/j.cub.2012.05.049) 22727698 PMC3418676

[14] Brittle A, Thomas C, Strutt D 2012 Planar polarity specification through asymmetric subcellular localization of Fat and Dachsous. Curr. Biol. 22, 907–914. (10.1016/j.cub.2012.03.053) 22503504 PMC3362735

[15] Saavedra P, Brittle A, Palacios IM, Strutt D, Casal J, Lawrence PA 2016 Planar cell polarity: the Dachsous/Fat system contributes differently to the embryonic and larval stages of *Drosophila*. Biol. Open. 5, 397–408. (10.1242/bio.017152) 26935392 PMC4890672

[16] Casal J, Struhl G, Lawrence PA 2002 Developmental compartments and planar polarity in *Drosophila*. Curr. Biol. 12, 1189–1198. (10.1016/s0960-9822(02)00974-0) 12176328

[17] Rovira M, Saavedra P, Casal J, Lawrence PA 2015 Regions within a single epidermal cell of *Drosophila* can be planar polarised independently. eLife 4, e06303 (10.7554/eLife.06303) PMC4341236 25671242

[18] Saavedra P, Vincent JP, Palacios IM, Lawrence PA, Casal J 2014 Plasticity of both planar cell polarity and cell identity during the development of *Drosophila*. eLife 3, e01569 (10.7554/eLife.01569) 24520160 PMC3918708

[19] Lawrence PA, Struhl G 1996 Morphogens, compartments, and pattern: lessons from *Drosophila*? Cell 85, 951–961. (10.1016/s0092-8674(00)81297-0) 8674123

[20] Blair SS 1995 Compartments and appendage development in *Drosophila*. BioEssays 17, 299–309. (10.1002/bies.950170406) 7741723

[21] Shimada Y, Yonemura S, Ohkura H, Strutt D, Uemura T 2006 Polarized transport of Frizzled along the planar microtubule arrays in *Drosophila* wing epithelium. Dev. Cell. 10, 209–222. (10.1016/j.devcel.2005.11.016) 16459300

[22] Harumoto T, Ito M, Shimada Y, Kobayashi TJ, Ueda HR, Lu B, Uemura T 2010 Atypical cadherins Dachsous and Fat control dynamics of noncentrosomal microtubules in planar cell polarity. Dev. Cell. 19, 389–401. (10.1016/j.devcel.2010.08.004) 20817616 PMC2951474

[23] Matis M, Russler-Germain DA, Hu Q, Tomlin CJ, Axelrod JD 2014 Microtubules provide directional information for core PCP function. eLife 3, e02893 (10.7554/eLife.02893) 25124458 PMC4151085

[24] Olofsson J, Sharp KA, Matis M, Cho B, Axelrod JD 2014 Prickle/spiny-legs isoforms control the polarity of the apical microtubule network in planar cell polarity. Development 141, 2866–2874. (10.1242/dev.105932) 25005476 PMC4197621

[25] Sharp KA, Axelrod JD 2016 Prickle isoforms control the direction of tissue polarity by microtubule independent and dependent mechanisms. Biol. Open. 5, 229–236. (10.1242/bio.016162) 26863941 PMC4810745

[26] Thurmond J et al. 2019 FlyBase 2.0: the next generation. Nucleic Acids Res. 47, D759–D765. (10.1093/nar/gky1003) 30364959 PMC6323960

[27] Sison CP, Glaz J 1995 Simultaneous confidence intervals and sample size determination for multinomial proportions. J. Am. Stat. Assoc. 90, 366–369. (10.1080/01621459.1995.10476521)

[28] Ferreira T, Hiner M, Rueden C, Miura K, Eglinger J, Chef B 2017 BAR 1.5.1. See 10.5281/zenodo.495245.

[29] Thevenaz P, Ruttimann UE, Unser M 1998 A pyramid approach to subpixel registration based on intensity. IEEE T. Image Process. 7, 27–41. (10.1109/83.650848) 18267377

[30] Meijering E, Dzyubachyk O, Smal I 2012 Methods for cell and particle tracking. Methods Enzymol. 504, 183–200. (10.1016/b978-0-12-391857-4.00009-4) 22264535

[31] R Core Team. 2019 R: a language and environment for statistical computing. Vienna, Austria: R Foundation for Statistical Computing.

[32] Fitak RR, Johnsen S 2017 Bringing the analysis of animal orientation data full circle: model-based approaches with maximum likelihood. J. Exp. Biol. 220, 3878–3882. (10.1242/jeb.167056) 28860118 PMC6514460

[33] Agostinelli C, Lund U. 2017 R package ‘circular': circular statistics (version 0.4-93). See https://r-forge.r-project.org/projects/circular/

[34] Signorell A 2019 multitple authors. DescTools: Tools for descriptive statistics. See https://cran.r-project.org/package=DescTools.

[35] Wickham H, François R, Henry L, Müller K 2019 dplyr: a grammar of data manipulation. See https://CRAN.R-project.org/package=dplyr

[36] Wickham H. 2016 Ggplot2: elegant graphics for data analysis. New York, NY: Springer-Verlag.

[37] Pruim R, Kaplan DT, Horton NJ 2017 The mosaic package: helping students to ‘Think with data’ using R. R J. 9, 77–102. (10.32614/RJ-2017-024)

[38] Ma D, Yang CH, McNeill H, Simon MA, Axelrod JD 2003 Fidelity in planar cell polarity signalling. Nature 421, 543–547. (10.1038/nature01366) 12540853

[39] Hale R, Brittle AL, Fisher KH, Monk NA, Strutt D 2015 Cellular interpretation of the long-range gradient of Four-jointed activity in the *Drosophila* wing. eLife 4, e05789 (10.7554/eLife.05789) PMC4338440 25707557

[40] Bosveld F et al. 2012 Mechanical control of morphogenesis by Fat/Dachsous/Four-jointed planar cell polarity pathway. Science 336, 724–727. (10.1126/science.1221071) 22499807

[41] Mao Y, Rauskolb C, Cho E, Hu WL, Hayter H, Minihan G, Katz FN, Irvine KD 2006 Dachs: an unconventional myosin that functions downstream of Fat to regulate growth, affinity and gene expression in *Drosophila*. Development 133, 2539–2551. (10.1242/dev.02427) 16735478

[42] Rogulja D, Rauskolb C, Irvine KD 2008 Morphogen control of wing growth through the Fat signaling pathway. Dev. Cell. 15, 309–321. (10.1016/j.devcel.2008.06.003) 18694569 PMC2613447

[43] Delon I, Chanut-Delalande H, Payre F 2003 The Ovo/Shavenbaby transcription factor specifies actin remodelling during epidermal differentiation in *Drosophila*. Mech. Dev. 120, 747–758. (10.1016/s0925-4773(03)00081-9) 12915226

[44] Walters JW, Dilks SA, DiNardo S 2006 Planar polarization of the denticle field in the *Drosophila* embryo: roles for Myosin II (zipper) and fringe. Dev. Biol. 297, 323–339. (10.1016/j.ydbio.2006.04.454) 16890930 PMC8711031

[45] Akhmanova A, Steinmetz MO 2008 Tracking the ends: a dynamic protein network controls the fate of microtubule tips. Nat. Rev. Mol. Cell Biol. 9, 309–322. (10.1038/nrm2369) 18322465

[46] Schuyler SC, Pellman D 2001 Microtubule ‘plus-end-tracking proteins’: the end is just the beginning. Cell 105, 421–424. (10.1016/s0092-8674(01)00364-6) 11371339

[47] Gomez JM, Chumakova L, Bulgakova NA, Brown NH 2016 Microtubule organization is determined by the shape of epithelial cells. Nat. Commun. 7, 13172 (10.1038/ncomms13172) 27779189 PMC5093320

[48] Fulford AD, McNeill H 2019 Fat/Dachsous family cadherins in cell and tissue organisation. Curr. Opin. Cell Biol. 62, 96–103. (10.1016/j.ceb.2019.10.006) 31739265

[49] Matis M, Axelrod JD 2013 Regulation of PCP by the Fat signaling pathway. Genes Dev. 27, 2207–2220. (10.1101/gad.228098.113) 24142873 PMC3814641

[50] Arata M, Sugimura K, Uemura T 2017 Difference in Dachsous levels between migrating cells coordinates the direction of collective cell migration. Dev. Cell. 42, 479–497e410. (10.1016/j.devcel.2017.08.001) 28898677

[51] Basler K, Struhl G 1994 Compartment boundaries and the control of *Drosophila* limb pattern by Hedgehog protein. Nature 368, 208–214. (10.1038/368208a0) 8145818

[52] Diaz-Benjumea FJ, Cohen SM 1995 Serrate signals through Notch to establish a Wingless-dependent organizer at the dorsal/ventral compartment boundary of the *Drosophila* wing. Development 121, 4215–4225. 8575321 10.1242/dev.121.12.4215

[53] Doherty D, Feger G, Younger-Shepherd S, Jan LY, Jan YN 1996 Delta is a ventral to dorsal signal complementary to Serrate, another Notch ligand, in *Drosophila* wing formation. Genes Dev. 10, 421–434. (10.1101/gad.10.4.421) 8600026

[54] Tabata T, Takei Y 2004 Morphogens, their identification and regulation. Development 131, 703–712. (10.1242/dev.01043) 14757636

[55] Struhl G, Barbash DA, Lawrence PA 1997 Hedgehog organises the pattern and polarity of epidermal cells in the *Drosophila* abdomen. Development 124, 2143–2154. 9187141 10.1242/dev.124.11.2143

[56] Ambegaonkar AA, Irvine KD 2015 Coordination of planar cell polarity pathways through Spiny-legs. eLife 4, e09946 (10.7554/eLife.09946) 26505959 PMC4764577

[57] Picone R, Ren X, Ivanovitch KD, Clarke JD, McKendry RA, Baum B 2010 A polarised population of dynamic microtubules mediates homeostatic length control in animal cells. PLoS Biol. 8, e1000542 (10.1371/journal.pbio.1000542) 21103410 PMC2982804

[58] Singh A, Saha T, Begemann I, Ricker A, Nusse H, Thorn-Seshold O, Klingauf J, Galic M, Matis M 2018 Polarized microtubule dynamics directs cell mechanics and coordinates forces during epithelial morphogenesis. Nat. Cell Biol. 20, 1126–1133. (10.1038/s41556-018-0193-1) 30202051

[59] Umetsu D, Aigouy B, Aliee M, Sui L, Eaton S, Julicher F, Dahmann C 2014 Local increases in mechanical tension shape compartment boundaries by biasing cell intercalations. Curr. Biol. 24, 1798–1805. (10.1016/j.cub.2014.06.052) 25065753

