# Supplementary material for: Planar cell polarity in the larval epidermis of Drosophila and the role of microtubules
Source: Open Biol. 2020 Dec 9;10(12):200290. doi: 10.1098/rsob.200290 (PMC7776564; doi:10.1098/rsob.200290)
Supplement: Supplementary figures [file rsob200290supp1.pdf]

Supplementary material of Pietra S, Ng KB, Lawrence PA, Casal J. 2020 Planar cell polarity in the larval epidermis of Drosophila and the role of microtubules. Open Biol. 10: 200290. https://doi.org/10.1098/rsob.200290

MOVIES

Movie 1. Film of microtubule dynamics in a representative larval A cell. EB1::GFP comets in a row 7 cell from the right hemisegment imaged for 4 minutes at 5.16s intervals. Juxtaposed movie shows manual tracing of 200 comet trajectories over the entire surface of the cell. Anterior is to the left, medial is down. Scale bar: 5μm.

Movie 2. Film of microtubule dynamics in a representative larval P cell. EB1::GFP comets in a row -1 cell from the left hemisegment imaged for 4 minutes at 5.16 s intervals. Juxtaposed movie shows manual tracing of 200 comet trajectories over the entire surface of the cell. Anterior is to the left, medial is up. Scale bar: 5μm.

SUPPLEMENTARY FIGURE LEGENDS

Figure S1. Quantitation of Ds levels at cellular interfaces in polarity modified larvae. (**A**) Dot plot, diagram of denticle polarity, and (**B**) pairwise comparisons are presented as in **figure 4**. Data are pooled from 3 images of larvae where overexpression of untagged Ds is specifically driven in tendons and changes the polarity of adjacent denticle cells (see **figure 2**). Ds distribution in (**C**) wild type (a detail from **figure 3A**) and (**D**) polarity modified larvae (*sr.Gal4 UAS.ectoDs*) is clearly different, reflecting the predicted changes in the landscape of Ds activity. For example in (**D**), more untagged Ds in T1 attracts more Ft molecules in row 2 cells to the T1/2 boundary, consequently displacing the row 2 endogenous, tagged Ds to the 2/3 boundary and raising fluorescence on that interface. The same effect emanating anteriorly from T2 raises Ds fluorescence at the 3/4 boundary. As expected, Ds amounts on the 2/3 and 3/4 boundaries are significantly higher than on the surrounding boundaries, arguing that the method is capable of detecting cellular interfaces with raised Ds activity. Attempts to relate observed polarity of a cell with the localisation of Ds at its membranes are compromised because we cannot determine how much Ds each of the two abutting cells is contributing to

their joint membrane. It is interesting to note that overexpressing ectoDs in the tendon cells has no significant effect on the amount of tagged Ds in 1/T1 or T2/5 boundaries. We think that is due to the several cells anterior to row 1 and the several cells posterior to row 6 dampening the effects. Scale bars: 20μm.

Figure S2. D localisation on limited parts of the plasma membrane. (**A**) Row 10 and 11 cells from a wildtype larva expressing *d::EGFP*, with cell outlines marked in magenta by DE-cad::tomato (see **figure 5C** for single EGFP channel). D is on just one side of each cell, but its localisation at the plasma membrane is not continuous: the row 10 cell accumulates D on the anterior membrane only where it confronts a T3 cell, not where it faces other row 10 cells; the row 11 cell has D localised at its posterior face, but only where it contacts row -2 cells. Scale bar: 10μm.

Figure S3. Unusual *ovo*-expressing clones with ambiguous polarity in row 11 cells. (**A,B**) Clones marked with EGFP and producing ectopic denticles in rows 10 and 11. *DE-cad::tomato* (magenta) labels cell boundaries and denticles, which in this area can be tenuous and hard to discern. (**A',B'**) Schemes of cell outlines and denticle orientation; denticles with uncharacteristic polarity are highlighted in red. (**A,A'**) Denticles pointing in opposite directions in two contiguous row 11 cells; all denticles in the neighbouring row 10 cells, point backwards. (**B,B'**) Denticles pointing in mixed directions within a single row 11 cell. Scale bars: 10μm.

Figure S4. Local polarity biases in microtubule growth. P values of chi-squared tests between numbers of comets whose orientation falls in opposite 22.5 degree sectors. Tables display the number of comets per sector and p values for larval and pupal sets of A and P cells. Sectors centred on the anteroposterior axis are highlighted in green.

Figure S5. Analysis of microtubule polarity in cells of the pupal abdomen, based on raw data kindly provided by the Axelrod group. (**A-E**) Rose diagrams of microtubule growth distribution, frequencies of comet orientation, and dot plot of microtubule direction in individual cells are presented as in **figure 8**. (**A,C,E**) Anterior pupal cells, (**B,D,E**) posterior pupal cells. n indicates total number of comets analysed, from the amount of pupae specified in parenthesis. Unlike ours, the data acquired by Axelrod's group contain no information

about which hemisegment they were sampled from; comet orientation is still classified as medial and lateral to facilitate comparison with our results, however these categories should be considered with caution. Note that, in contrast with larval data where differences between the frequencies of comets in opposite quadrants are very weak (**figure 8C,D**), in pupae there are significant biases in the proportion of anteroposteriorly and mediolaterally growing microtubules (see non-overlapping confidence intervals in **C** and **D**).

Figure S6. Maximum likelihood best models of microtubule angular distributions. Using a maximum likelihood approach [**34**] we plot the angular distribution of all growing microtubules and the best fit is to bimodal distributions with two peaks near 180 degrees apart in the mediolateral axis. The distribution densities are shown in blue (darker blue representing the anterior and posterior 90 degree quadrants). A circular histogram (bin size 22.5 degree) of the angle data is at the centre of each plot in grey. The mean vector is shown in red and the two mean angles are shown with discontinuous arrows. The mean values (θ), concentration parameters (κ), proportional size of the first distribution (λ), mean vector angle ($\bar{\theta}$) and dispersion ($\bar{R}$) are shown below each plot. A deviation of 10 degrees in one of the peaks of the distribution from the true mediolateral axis is enough to create a difference in the density area of the anterior and posterior quadrants. In both larval and pupal sets of A cells the area of the posterior quadrant density closest to the deviated peak is slightly bigger (red arrowhead) than the anterior one (green arrowhead). In both larval and pupal sets of P cells the area of the anterior quadrant is slightly bigger (red arrowhead) than the posterior one (green arrowhead).

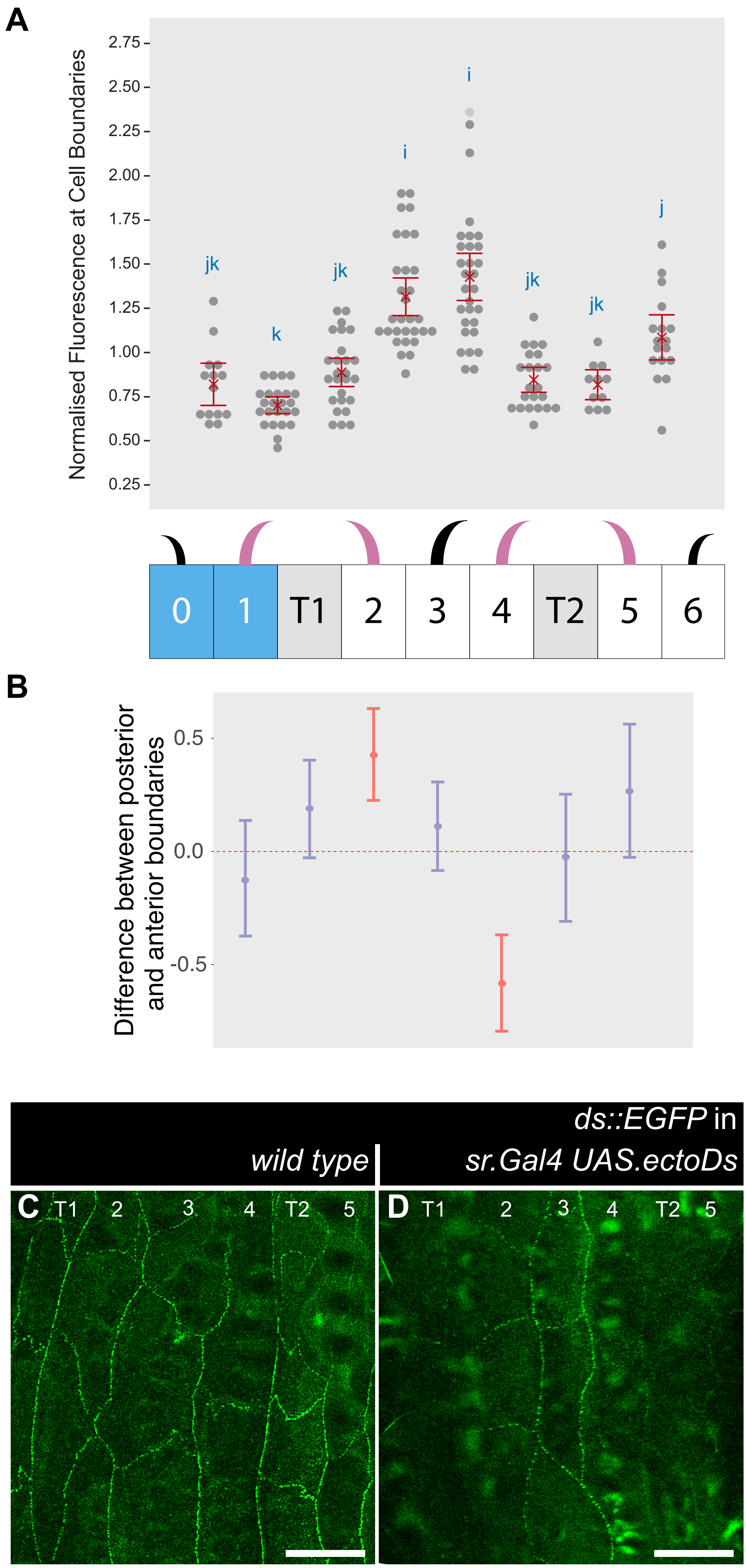

Pietra et al. Figure S1

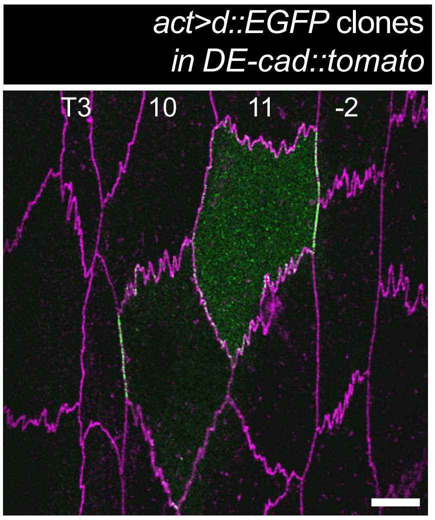

Pietra et al. Figure S2

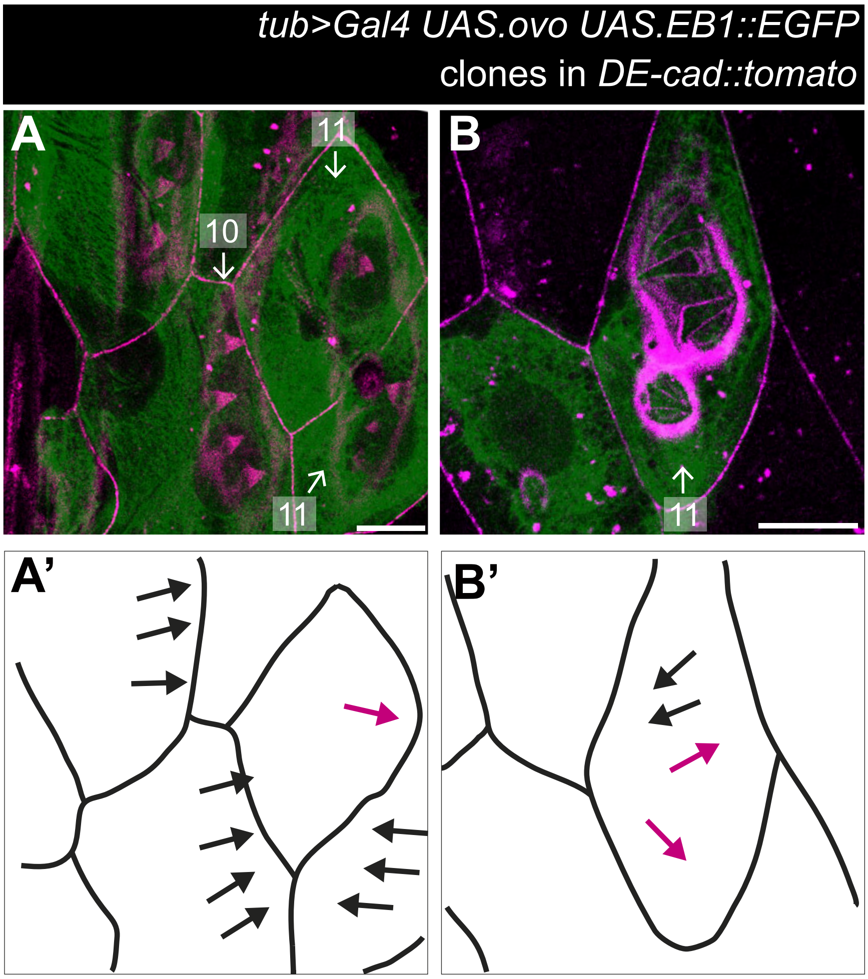

Pietra et al. Figure S3

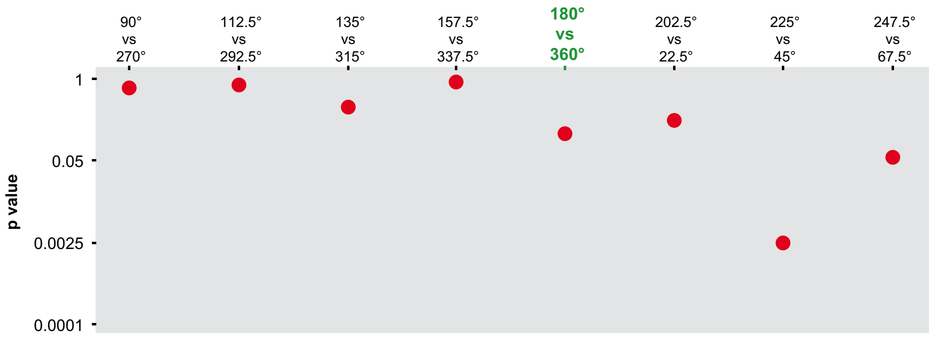

A larval cells

Sectors	Number of comets		p value
90° vs 270°	195	188	0.72
112.5° vs 292.5°	175	170	0.79
135° vs 315°	110	124	0.36
157.5° vs 337.5°	99	97	0.89
**180° vs 360°**	**56**	**73**	**0.13**
202.5° vs 22.5°	73	59	0.22
225° vs 45°	79	122	0.002
247.5° vs 67.5°	124	156	0.06

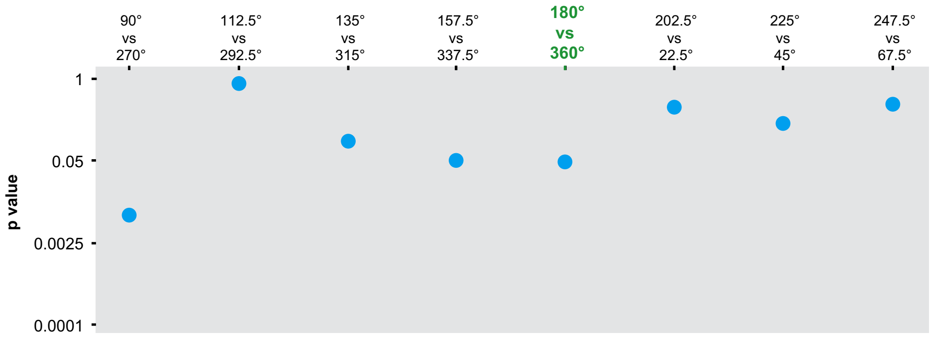

P larval cells

Sectors	Number of comets		p value
90° vs 270°	215	275	0.007
112.5° vs 292.5°	189	185	0.84
135° vs 315°	121	97	0.10
157.5° vs 337.5°	74	52	0.05
**180° vs 360°**	**86**	**62**	**0.05**
202.5° vs 22.5°	62	52	0.35
225° vs 45°	96	79	0.20
247.5° vs 67.5°	160	145	0.39

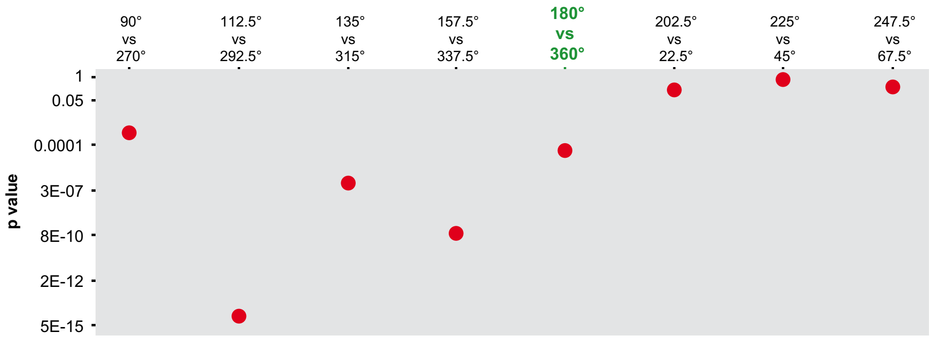

A pupal cells

Sectors	Number of comets		p value
90° vs 270°	658	789	0.0006
112.5° vs 292.5°	303	524	1.53E-14
135° vs 315°	180	287	7.37E-07
157.5° vs 337.5°	123	239	1.08E-09
**180° vs 360°**	**143**	**220**	**0.00005**
202.5° vs 22.5°	208	235	0.20
225° vs 45°	271	279	0.73
247.5° vs 67.5°	396	427	0.28

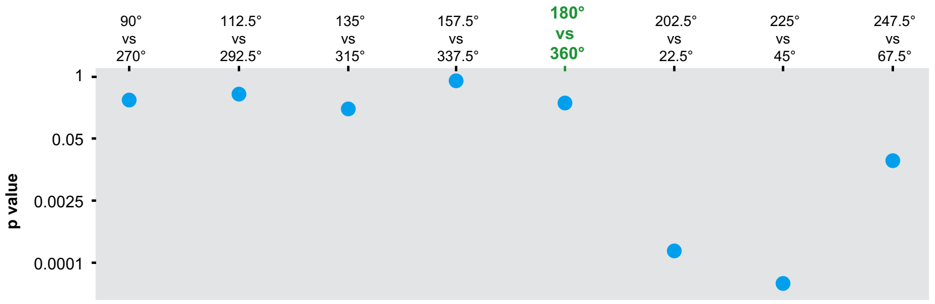

P pupal cells

Sectors	Number of comets		p value
90° vs 270°	299	323	0.34
112.5° vs 292.5°	117	129	0.44
135° vs 315°	55	69	0.21
157.5° vs 337.5°	56	54	0.85
**180° vs 360°**	**67**	**55**	**0.28**
202.5° vs 22.5°	86	44	0.0002
225° vs 45°	119	64	0.00005
247.5° vs 67.5°	155	116	0.02

Pietra et al. Figure S4

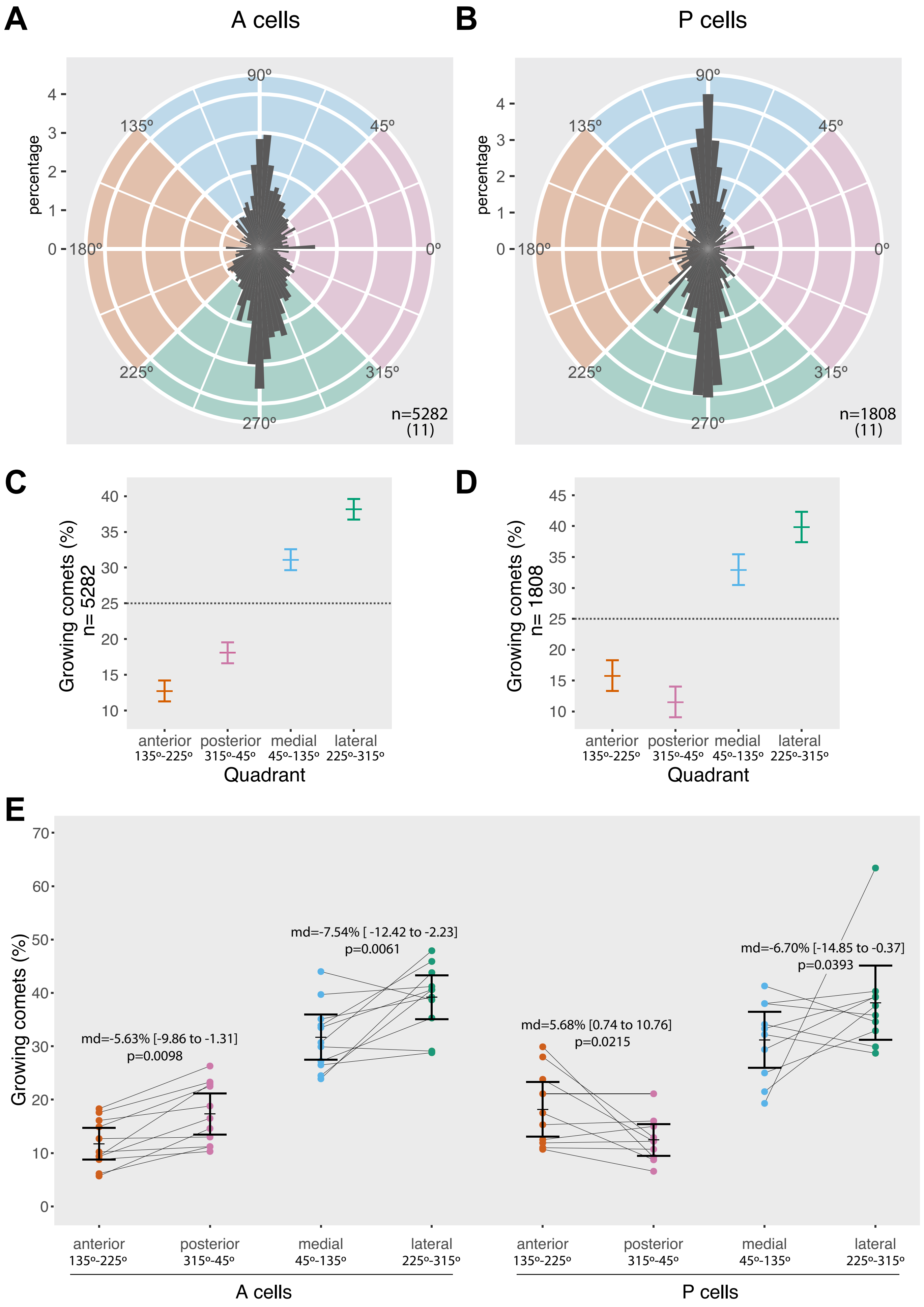

Pietra et al. Figure S5

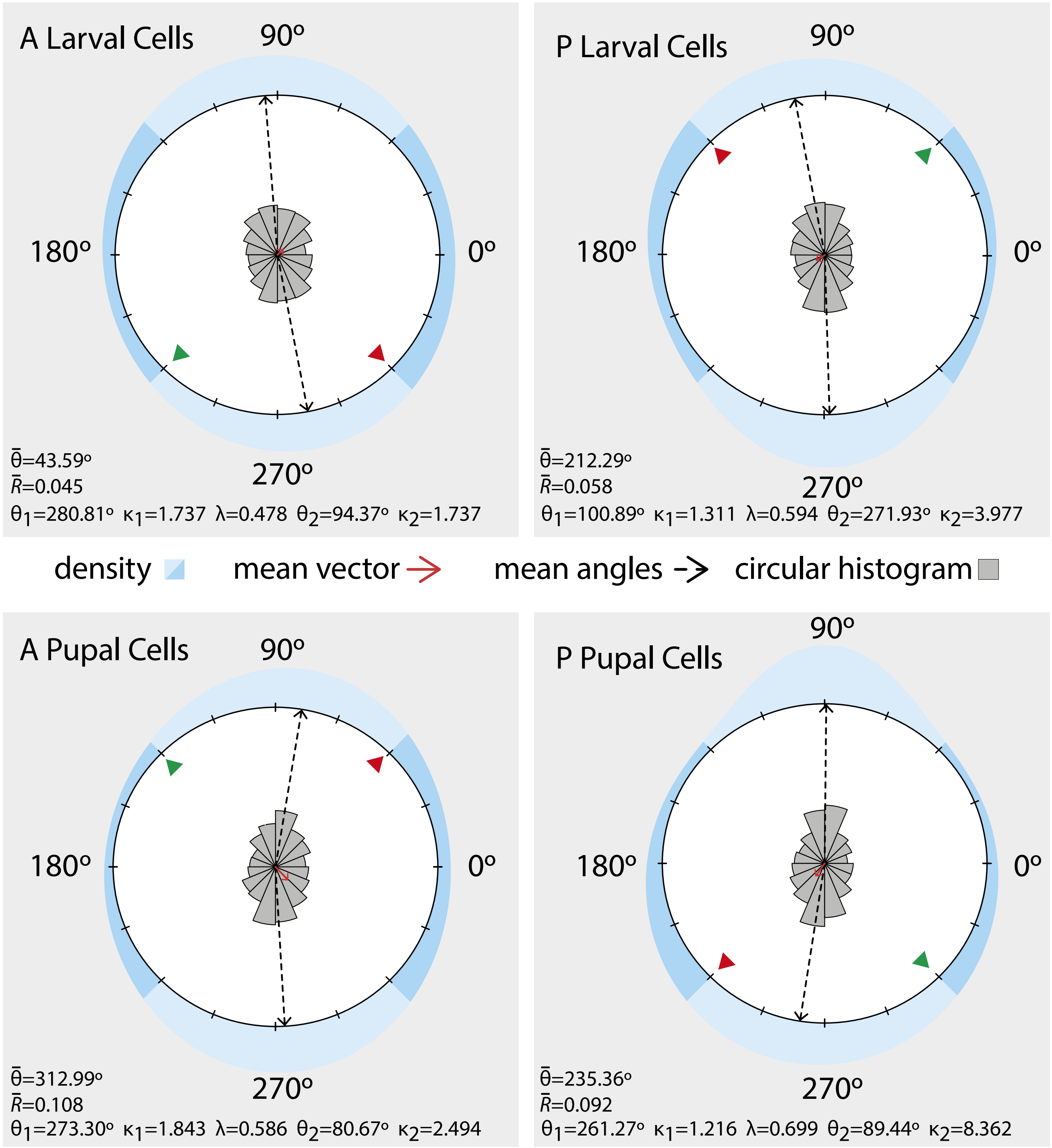

Pietra et al. Figure S6
